# Magnetic resonance imaging-based simplified MaRIA scores are associated with future surgery in Crohn’s disease, but modest correlation with ileo-colonoscopic inflammation limits their utility in clinical trials: results from the PROFILE trial

**DOI:** 10.1093/ecco-jcc/jjag056

**Published:** 2026-05-19

**Authors:** Megha Bhandari, Mohmmed Tauseef Sharip, Nurulamin M Noor, Samir Khwaja, Hania Paverd, Katja N De Paepe, Edmund Godfrey, Katy Hickman, Kamal V Patel, Sreedhar Subramanian, Tim Raine, James C Lee, Nicholas A Kennedy, Sara Upponi, Miles Parkes, PROFILE Study Group, Klaartje Bel Kok, Shahida Din, Alan Steel, John Gordon, Shanika de Silva, Melissa Smith, Matthew Johnson, Alexandra Kent, Rebecca Saich, Tariq Ahmad, Dharmaraj Durai, Gordon Moran, Alan Wiles, Pritash Patel, Paul Banim, Rachel Cooney, Peter Irving, Dan Sharpstone, James Berrill, Craig Mowat, Horace Williams, Phil Harvey, Rhys Butcher, Sean Weaver, Rakesh Chaudhary, Anjan Dhar, Arvind Ramadas, Emma Wesley, Robert Boulton-Jones, Jeffrey Butterworth, Rishi Goel, Ailsa Hart, Babur Javaid, Paul Knight, Sam Powles, Richard Ally Speight, Andy Cole, Andy Li, Francis Dowling, Suhaylah Bhatti, Biljana Brezina, Juan De La Revilla Negro, James Lindsay

**Affiliations:** Department of Gastroenterology, Cambridge University Hospitals NHS Foundation Trust, Cambridge, CB2 0QQ, United Kingdom; Department of Medicine, University of Cambridge School of Clinical Medicine, Cambridge, CB2 0AW, United Kingdom; Department of Gastroenterology, Cambridge University Hospitals NHS Foundation Trust, Cambridge, CB2 0QQ, United Kingdom; Department of Medicine, University of Cambridge School of Clinical Medicine, Cambridge, CB2 0AW, United Kingdom; Department of Gastroenterology, Cambridge University Hospitals NHS Foundation Trust, Cambridge, CB2 0QQ, United Kingdom; Department of Medicine, University of Cambridge School of Clinical Medicine, Cambridge, CB2 0AW, United Kingdom; Department of Radiology, Cambridge University Hospitals NHS Foundation Trust, Cambridge, CB2 0QQ, United Kingdom; Department of Radiology, Cambridge University Hospitals NHS Foundation Trust, Cambridge, CB2 0QQ, United Kingdom; Department of Radiology, Beth Israel Deaconess Medical Center and Harvard Medical School, Boston, MA 02215, United States; Department of Radiology, Cambridge University Hospitals NHS Foundation Trust, Cambridge, CB2 0QQ, United Kingdom; Department of Radiology, Cambridge University Hospitals NHS Foundation Trust, Cambridge, CB2 0QQ, United Kingdom; Department of Gastroenterology, St George’s University Hospitals NHS Foundation Trust, London, SW 17 0QT, United Kingdom; Department of Gastroenterology, Cambridge University Hospitals NHS Foundation Trust, Cambridge, CB2 0QQ, United Kingdom; Department of Gastroenterology, Cambridge University Hospitals NHS Foundation Trust, Cambridge, CB2 0QQ, United Kingdom; Genetic Mechanisms of Disease Laboratory, The Francis Crick Institute, London, NW1 1AT, United Kingdom; Department of Gastroenterology, UCL Institute for Liver and Digestive Health, Royal Free Hospital, London, NW3 2QG, United Kingdom; Department of Gastroenterology, Royal Devon University Healthcare NHS Foundation Trust, Exeter, EX2 5DW, United Kingdom; Department of Radiology, Cambridge University Hospitals NHS Foundation Trust, Cambridge, CB2 0QQ, United Kingdom; Department of Gastroenterology, Cambridge University Hospitals NHS Foundation Trust, Cambridge, CB2 0QQ, United Kingdom; Department of Medicine, University of Cambridge School of Clinical Medicine, Cambridge, CB2 0AW, United Kingdom

**Keywords:** Crohn’s disease, clinical trial, colonoscopy, MRI, endpoints, inflammation, ulceration, surgery, endoscopic remission

## Abstract

**Background:**

Ileo-colonoscopy provides accurate assessment of mucosal inflammation in Crohn’s disease. Magnetic resonance imaging (MRI) offers a non-invasive alternative. MRI-based scores such as the simplified MaRIA (sMaRIA) have been proposed for measuring treatment response in clinical trials, but systematic comparisons with endoscopic scoring systems in prospective datasets are lacking.

**Methods:**

PROFILE trial participants underwent ileo-colonoscopy and small bowel MRI at baseline and week 48 (end-of-trial). Using this dataset our primary objective was to assess the correlation between Simple Endoscopic Score for Crohn’s Disease (SES-CD) and sMaRIA scores using contemporaneous ileo-colonoscopy and MRI studies. We also assessed the utility of sMaRIA as a clinical trial endpoint, and correlation between baseline sMaRIA and need for future surgery. Endoscopic and MRI remission were defined as SES-CD and sMaRIA scores of 0. Ulcer subscores were also examined.

**Results:**

In total, :285 patients had paired baseline ileo-colonoscopy and MRI studies and 220 had paired end-of-trial studies—undertaken a median of 35 and 22 days apart respectively. Correlation between total SES-CD and sMaRIA scores on end-of-trial studies was moderate (Spearman’s ρ = 0.428). MRI/sMaRIA identified only 18/143 ulcerated segments seen on ileo-colonoscopy. While remission rates between PROFILE treatment arms were significantly different on ileo-colonoscopy/SES-CD (*P* = .005), this difference was not seen on MRI/sMaRIA (*P* = .564). Patients requiring abdominal surgery had higher baseline sMaRIA scores than those who did not (*P* = .015), while SES-CD scores were not different between these groups (*P* = .830).

**Conclusions:**

MRI should be considered as complementary to ileo-colonoscopy for assessing ileal Crohn’s disease. sMaRIA should not replace SES-CD in clinical trials.

## 1. Introduction

Crohn’s disease is characterized by patchy transmural inflammation that can affect any part of the gastrointestinal (GI) tract, but where the ileum or colon or both are most commonly involved. The first-line investigation of choice for assessing disease activity has conventionally been ileo-colonoscopy, which enables direct visualization of the mucosa, detailed assessment of inflammatory activity, and collection of tissue biopsies. However, ileo-colonoscopy is invasive, can be uncomfortable for patients, and carries potential procedural risks. Given these limitations, there is growing interest in alternative non-invasive imaging modalities for disease assessment and evaluation of treatment response. Magnetic resonance imaging (MRI) can evaluate transmural inflammation as well as strictures and fistulae without the ionizing radiation associated with computed tomography (CT) scans.^1^

In clinical trials, ileo-colonoscopy assessment of Crohn’s disease inflammatory activity with centrally read assessment of videos is the current gold standard, as recognized by regulators such as the Food and Drug Administration (FDA) and European Medicines Agency (EMA).^2^^,^^3^ The Simple Endoscopic Score for Crohn’s Disease (SES-CD) is a validated instrument for quantifying inflammation observed during ileo-colonoscopy and is widely used as an endpoint in clinical trials.^4^

It is currently unknown whether MRI could be used as an alternative to ileo-colonoscopy in clinical trials to assess disease activity and treatment response.^5^^,^^6^

Several scoring systems have been developed to standardize the quantitation of Crohn’s disease activity using MRI, with the Magnetic Resonance Index of Activity (MaRIA) score among the first to be validated.^7^^,^^8^ However, the MaRIA score is complex to calculate and requires the use of intravenous gadolinium to evaluate enhancement, so a simplified version (sMaRIA) was introduced. This does not require contrast and correlates well with MaRIA scores.^9^ sMaRIA evaluates the absence or presence of wall thickening, edema, fat stranding, and ulceration across the same five ileo-colonic segments as SES-CD.^10^ Given its greater simplicity, the sMaRIA score has been proposed as potentially more useful.^11^

Several reports have suggested a correlation between MaRIA or sMaRIA and SES-CD scores.^9^^,^^12^ However, most reported studies were conducted retrospectively and carried significant risk of selection bias, given that MRI scans are usually requested in patients with more severe or treatment-refractory disease to evaluate for potential complications.^12^ To date, there have been no large, prospective studies comparing MRI and ileo-colonoscopy-based scoring systems to evaluate treatment response in the context of a Crohn’s disease clinical trial. Here we report findings from the PROFILE trial dataset, the largest cohort of paired MRIs and ileo-colonoscopies to date.

## 2. Methods

We undertook this study as a pre-specified analysis of the PROFILE trial, for which primary and secondary outcome results have been previously published.^13^ Our primary objective in the current study was to assess the correlation between sMaRIA and SES-CD scores, based on contemporaneous MRI and ileo-colonoscopy studies undertaken in PROFILE. The specific aims were to determine: (1) how the sMaRIA score correlated with SES-CD in detecting and quantifying inflammation and ulceration in Crohn’s disease—both in terms of total scores and subscores stratified by disease location, specifically for the terminal ileum; (2) how sMaRIA performed compared to SES-CD if used as an endpoint in a clinical trial; and (3) whether baseline sMaRIA or SES-CD scores were associated with the development of Crohn’s disease complications requiring abdominal surgery.

### 2.1. Participants

The study cohort consisted of adult patients who had been newly diagnosed with Crohn’s disease at the point of PROFILE trial enrolment.^13^ Notably, inclusion criteria for PROFILE required evidence of active disease in terms of biomarkers (raised C-reactive protein [CRP] [>upper limit of normal] or raised calprotectin [>200 µg/g] or both) and endoscopic inflammation. During the trial, patients were randomized to receive either “top-down” therapy with combination infliximab and immunomodulator from enrolment or to “accelerated step-up” therapy, with sequential addition of immunomodulator and then infliximab in response to disease flares.^13^

### 2.2. Ileo-colonoscopy/SES-CD and MRI/sMaRIA scoring

PROFILE trial participants underwent ileo-colonoscopy before the start and at the end of the 48-week trial period. Twelve consultant gastroenterologists, who underwent prior training and validation,^14^ centrally reported SES-CD scores in PROFILE.^13^ After undergoing the consensus training exercise, all ileo-colonoscopies were centrally read by a single gastroenterologist, blinded to clinical data and treatment allocation. Ten per cent of these videos were assigned to a second blinded central reader to check for consistency and maintenance of inter-reader reliability.

Participants also underwent small bowel MRI using a standardized protocol around the time of enrolment (MRI findings were not a required inclusion criterion, whereas endoscopic inflammation was) and at week 48. All MRI images were acquired using either 1.5- or 3.0-T MRI scanners. Further details on the MRI protocol used in the PROFILE trial are provided (Supplementary Appendix). Five consultant GI radiologists, who underwent prior training and validation, centrally reported sMaRIA scores on all MRI scans.^15^ Notably, the central reader development protocol encompassed independent scoring on a pool of MRI scans, inter-reader reliability assessment using intraclass correlation coefficient (ICC), radiology consensus meeting, and iterative rounds of scoring on further MRI scans and evaluation until satisfactory inter-reader reliability was achieved. After undergoing this consensus exercise all scans were centrally read by a single radiologist, with 10% of these scans assigned to a second blinded central reader to check for consistency and maintenance of inter-reader reliability. All GI radiologists were blinded to the corresponding ileo-colonoscopy results, clinical data, and treatment allocation. A score was derived for each bowel segment and summed to calculate a global sMaRIA score out of a total of 25.^15^

### 2.3. Statistical analysis and outcomes

Statistical methods including Spearman’s rank correlation were used to assess the strength and direction of association between SES-CD and sMaRIA scores, given the ordinal nature of values, with a coefficient close to 1 indicating a strong positive correlation and 0 being no correlation. McNemar’s test was used to compare ulcer detection between MRI and ileo-colonoscopy at the start and end of the trial. Significance was defined as *P* < .05. Data were analysed using the Python 3.12.1 packages numpy, pandas, and matplotlib.

Both SES-CD and sMaRIA scores categorize inflammatory activity as inactive, mild, moderate, or severe (Table S1).^9^ To compare the association between findings at baseline of the two modalities and future need for surgery, patients were stratified into two subgroups. Subgroup 1 comprised patients with ileo-colonoscopy evidence of inflammation at baseline but a normal MRI (index sMaRIA score = 0), while subgroup 2 included patients with evidence of active inflammation on both ileo-colonoscopy and MRI (index sMaRIA score > 0). Comparisons between these groups were performed using chi-squared tests or Fisher’s exact test where appropriate.

Disease behavior at baseline in the PROFILE trial had been categorized by local clinicians as Montreal classification B1/B2/B3 using clinical, endoscopic, and (where available) imaging data. Reflecting clinical practice, MRI findings were not available until some time after diagnosis for a high proportion of patients. We explored how availability of MRI data led to re-classification compared to clinical and endoscopic data alone.

In this study, we defined endoscopic remission as a normal ileo-colonoscopy (total SES-CD score = 0) and radiological remission as normal MRI (total sMaRIA score = 0), consistent with recent global consensus recommendations.^16^ The original PROFILE trial endoscopic outcome was based on an SES-CD ulcer subscore of 0. To compare ulcer-free remission as identified by ileo-colonoscopy and MRI we here used both this original endoscopic definition (SES-CD ulcer subscore = 0) and a less stringent definition (SES-CD ulcer subscore ≤ 1), thereby allowing for the presence of aphthous ulceration, which it might be anticipated would be difficult to detect on MRI. These endoscopic ulcer sub-scores were compared to an MRI-derived sMaRIA ulcer score of 0, which required absence of ulceration detected on MRI when scored using sMaRIA.

### 2.4. Ethical considerations

Patients were enrolled in 40 hospitals across the UK. The Cambridge South research ethics committee approved the protocol (number 17/EE/0382), and the trial followed Good Clinical Practice guidelines.

## 3. Results

In our study 298 patients had paired MRIs and ileo-colonoscopies at baseline. Thirteen MRIs were excluded due to poor image quality, leaving 285 paired baseline studies available for analysis. The baseline ileo-colonoscopy had been performed before trial enrolment and MRI undertaken a median of 35 days after this. All patients received a tapering course of corticosteroid following enrolment and were randomized to either “top-down” infliximab and immunomodulator combination therapy or to “accelerated step-up” care. At the end of the 48-week trial, 253 patients had paired MRI and ileo-colonoscopy data collected. Of these, 33 MRIs were excluded due to poor image quality, and therefore 220 end-of-trial paired MRIs and ileo-colonoscopies were available for analysis. There was a median time difference of 21.5 days between end-of-trial studies, during which treatments did not change.

### 3.1. Correlation between SES-CD and sMaRIA

**Figure 1.**
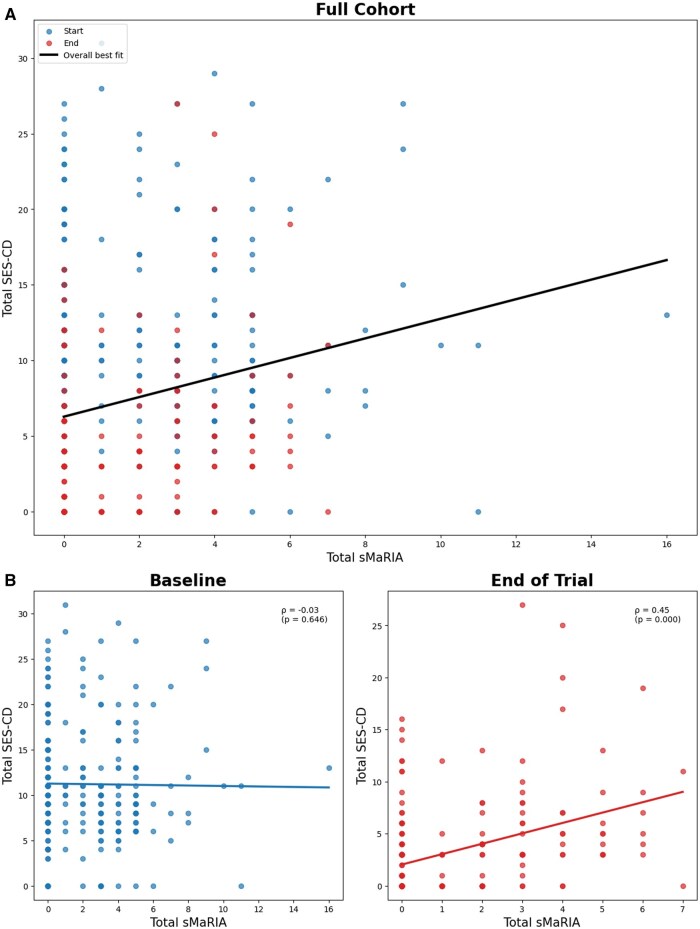
(A) Correlation between total sMaRIA and Simple Endoscopic Score for Crohn’s Disease (SES-CD) scores for all PROFILE participants and (B) correlation between total sMaRIA and SES-CD scores at start and end-of-trial. Spearman’s ρ assesses the association between the two variables, with values closer to ±1 indicating a stronger correlation. *P*-values reflect the statistical significance of the observed association.

Comparing total sMaRIA scores versus total SES-CD scores at the start of the trial, we observed a Spearman’s rank correlation coefficient of −0.031 (no correlation). As this lack of correlation could be attributed to the greater time difference between ileocolonscopy and MRI at baseline and the dynamic changes in treatment at this time, all subsequent analyses comparing the utility of SES-CD and sMaRIA for assessing Crohn’s disease activity focused on the end-of-trial (week 48) procedures only. The Spearman’s rank correlation coefficient at week 48 was 0.446 (moderate positive correlation) (Figure 1).

When focusing on a binary classification of inactive versus active disease, we found a moderate correlation between SES-CD and sMaRIA at week 48 (phi coefficient ϕ = 0.40, *P* < .0001) (Table S1). Of the 129 patients classified as having inactive disease by sMaRIA, 41 were noted to have active disease on their ileo-colonoscopy—with 29, 10, and two respectively classified as having mild, moderate, or severe inflammation based on their SES-CD scores (Table 1;  Table S2). At the other end of the spectrum, eight of 220 patients were classified by SES-CD as having severe disease at week 48, whereas sMaRIA identified 53 of 220 patients as severe. Among these 53 patients, ileo-colonoscopic assessment concurred in five cases; the remaining patients were classified by SES-CD as having inactive disease in nine cases, mild disease in 25 cases, and moderate disease in 14 cases.

**Table 1.** Confusion matrix comparing performance of sMaRIA and Simple Endoscopic Score for Crohn’s Disease (SES-CD) for different categories of inflammatory activity at week 48.

SES-CD sMaRIA	Inactive	Mild	Moderate	Severe	Total
**Inactive**	88	7	9	9	113
**Mild**	29	6	9	25	69
**Moderate**	10	2	4	14	30
**Severe**	2	0	1	5	8
**Total**	129	15	23	53	220

**Figure 2.**
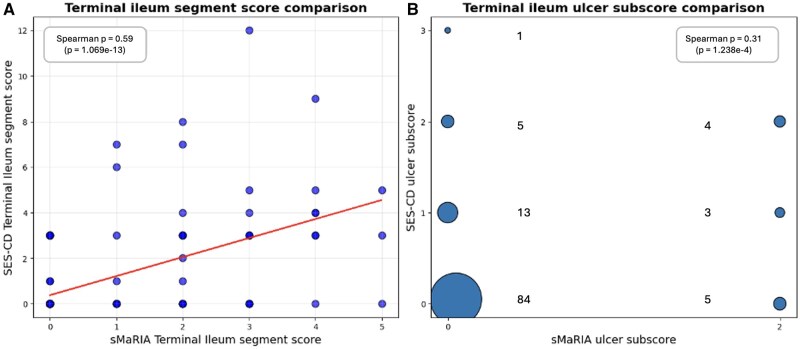
(A) Correlation between terminal ileal segment sMaRIA and Simple Endoscopic Score for Crohn’s Disease (SES-CD) scores and (B) dot plot showing correlation between terminal ileal ulcer component of sMaRIA and SES-CD scores. The dot size is proportional to the number of patients contributing to each coordinate.

When assessing correlation stratified by disease location, the highest correlation was noted to be for the ileal-only disease phenotype (Table S3). To enable a fair comparison between SES-CD (where the scoring system is dominated by colonic segments) and sMaRIA (which primarily scores small bowel segments) we next focused on terminal ileal scores in the subset of patients with terminal ileal involvement either on ileo-colonoscopy or MRI or both. Total terminal ileal sMaRIA and SES-CD scores showed moderate correlation (Spearman’s rank correlation coefficient = 0.59; Figure 2A), but assessment for the presence or absence of ulceration in the terminal ileum showed only weak correlation between the modalities (Spearman’s rank correlation coefficient = 0.31; Figure 2B).

Subsequently, ulcer detection across all bowel segments was assessed. McNemar’s test revealed a significant discordance between paired MRI ulcer detection and ileo-colonoscopy findings (*P* < .001). Comparing ulcer detection by SES-CD versus sMaRIA across 1100 segments in 220 patients at the end-of-trial assessments: by ileo-colonoscopy 50 segments had ulcers > 5 mm (SES-CD ulcer subscore > 1) and 93 segments had ulcers 1–5 mm in size (SES-CD ulcer subscore = 1). Of these just 9/50 and 9/93 respectively (total 18/143) were detected by MRI. The remaining 957 segments showed no ulceration on ileo-colonoscopy (SES-CD ulcer subscore = 0).

We then performed an additional analysis comparing specific features of the sMaRIA score to the SES-CD score (Table S4). We found that mural edema and wall thickening demonstrated the strongest overall correlation with SES-CD scores (mean *r* = 0.301 and 0.294 respectively), compared to fat stranding (*r* = 0.210) and ulcer detection (*r* = 0.172). Mural edema showed particular reliability in the terminal ileum (*r* = 0.47) but with less reliability in the colon.

### 3.2. Assessing need for surgery stratified by MRI findings at baseline

**Figure 3.**
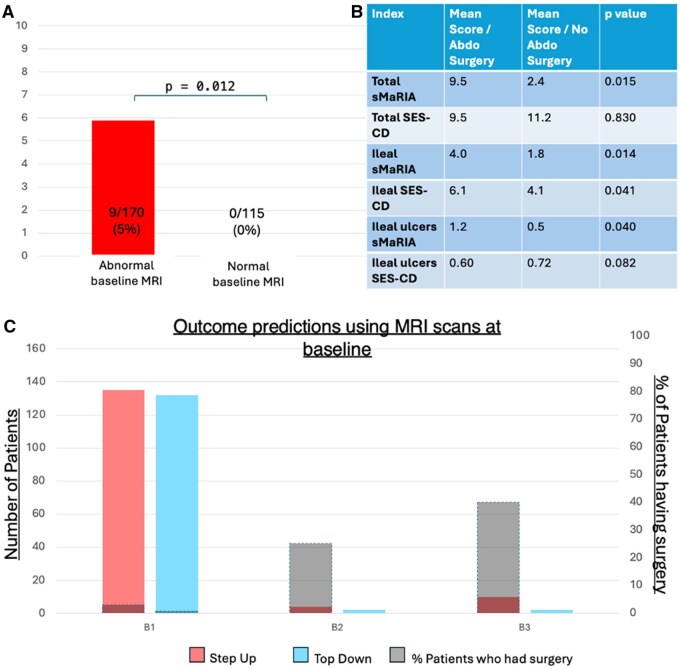
(A) Need for abdominal surgery by week 48 of the PROFILE trial based on abnormal versus normal baseline magnetic resonance imaging (MRI) and (B) comparison of scores and score components in patients who went on to require surgery versus those who did not and (C) percentage of patients who went on to have surgery, stratified by disease phenotype classification when MRI is used to inform disease behaviour. B1, inflammatory; B2, stricturing; B3, penetrating.

We next compared the need for surgery between patients who had inflammation evident on their baseline MRI (subgroup 1, *n* = 179) versus those whose baseline MRI appeared normal (subgroup 2, *n* = 115). All nine patients requiring abdominal surgery during the 48-week PROFILE trial were in the former group (Fisher’s exact test, *P* = .012; Figure 3A). Seven out of these nine patients were initially classified by local teams at baseline as having inflammatory (Montreal B1) Crohn’s disease, based on clinical findings (history, examination, bloods, and ileo-colonoscopy). Following review of the index MRI, which took place after the “baseline” consultation, three of these seven patients were re-classified as having imaging evidence of penetrating (B3) disease. All three patients were noted to have markedly raised CRP levels ranging from 38 to 73 mg/L (Table S5). Additionally, two out of the nine patients requiring abdominal surgery had stricturing (B2) disease from diagnosis (Table S5).

Mean total sMaRIA scores were higher in patients who went on to require abdominal surgery compared to those who did not (9.5 vs 2.4; *P* = .015), but mean total baseline SES-CD scores did not differ (9.5 vs 11.2; *P* = .830) (Figure 3B). Patients who required abdominal surgery in the first year after diagnosis also had higher mean ileal ulcer scores on sMaRIA compared to those who did not need abdominal surgery, whereas there was no difference in SES-CD ileal ulcer scores (Figure 3B).

In some instances clinical classification of disease behavior (based on history, examination, endoscopy, and blood tests) at enrolment to PROFILE had to be revised in light of MRI findings. Reclassification went in both directions (ie, clinical B2 reclassified as radiological B1—and clinical B1 reclassified as radiological B2 or B3). In the step-up arm, 4/12 patients initially classified as B1 disease but with B2 or B3 disease evident on their baseline MRI subsequently required surgery over the next 48 weeks (Table S6). Conversely, in the top-down group, none of the patients who had B2 or B3 disease evident at index MRI needed abdominal surgery over the next 48 weeks (Figure 3C).

### 3.3. sMaRIA as a clinical trial endpoint

**Figure 4.**
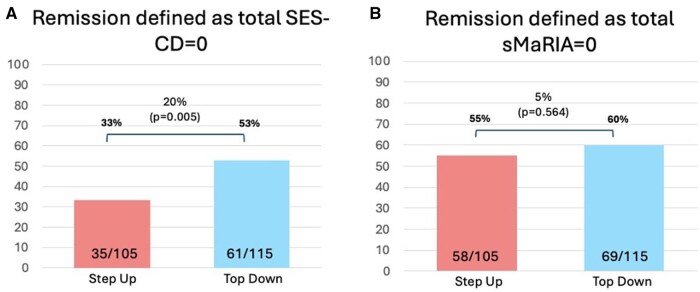
PROFILE clinical trial endpoints using (A) endoscopic remission defined by total Simple Endoscopic Score for Crohn’s Disease (SES-CD) score of 0 and (B) magnetic resonance imaging (MRI)-defined remission defined by total sMaRIA score 0.

Finally, we investigated the potential use of sMaRIA as a clinical trial endpoint to provide an objective evaluation of treatment response. Defining endoscopic remission as a total SES-CD of 0 (normal endoscopy), we observed in PROFILE a difference of 20% between the “accelerated step-up” and “top-down” treatment arms (33% [35/105] vs 53% [61/115] respectively; *P* = .005, Figure 4A). However, using an sMaRIA score of 0 as the remission definition, we saw no difference between the groups (55% [58/105] following “accelerated step-up” vs 60% [69/115] following “top-down” treatment; *P* = .564, Figure 4B).

The original PROFILE trial endoscopic outcomes focused on ulcer-free remission rather than the total SES-CD score. Whether taking the strict definition of no ulcers (SES-CD ulcer sub-score = 0), as used in the PROFILE trial, or allowing aphthous ulcers (SES-CD ulcer sub-score ≤ 1) within the definition, endoscopic remission was higher in patients receiving “top-down” versus “accelerated step-up” treatment (Figure S1A and B). Using SES-CD ulcer sub-score ≤ 1, remission rates were 68% [78/115] versus 47% [49/105] respectively (*P* = .001). In contrast, uniformly high levels of sMaRIA-defined ulcer-free remission were observed in both treatment arms (“top-down” 94% [108/115] vs “accelerated step-up” 87% [91/105]), with no significant difference between them (*P* = .110, Figure S1C).

## 4. Discussion

In our study, we observed a dissociation between MRI-based and endoscopy-based assessments of disease activity in Crohn’s disease. Our findings highlight the respective strengths and weaknesses of MRI and ileo-colonoscopy and have important implications for clinical practice and clinical trials, as well as identifying a potential role for MRI in surgical risk stratification. While MRI is recognized for its utility in investigating transmural Crohn’s disease complications, including potential small bowel strictures and fistulae,^17^^,^^18^ we observed that sMaRIA had only moderate correlation with SES-CD for detection of active mucosal inflammation and ulceration. This was true even after excluding paired studies done at baseline in the PROFILE trial, where there was a significant time-lag and dynamic treatment change between ileo-colonoscopy and MRI (Figures 1 and 2; Table 1).

There are likely to be several factors that help explain the relatively modest correlation between sMaRIA and SES-CD that we found in PROFILE compared to some previously reported studies.^8^^,^^12^ Among the most important is the method of patient ascertainment and associated differences in clinical characteristics. Previous studies demonstrating good concordance recruited participants referred for imaging based on clinical need. Such patients would generally have had more severe and long-standing disease, with a higher prior probability of radiological abnormality being detected. Furthermore, most previous studies assessing response to biologic therapy included patients where treatment had been initiated years or even decades after diagnosis, a large proportion of whom were non-responders at the time of imaging assessment. Many patients undergoing end-of-trial MRI scans in PROFILE, by contrast, were in remission or had only mild disease activity—and had no more than a 1-year history of Crohn’s disease.

Putting our study in context, METRIC was the largest previously published study investigating the diagnostic accuracy of MRI in Crohn’s disease.^16^ It included 284 patients and suggested broadly comparable diagnostic accuracy between MRI and intestinal ultrasound (IUS). However, METRIC did not mandate ileo-colonoscopy assessment for comparison and was not designed to assess treatment response.

While the presence of endoscopic mucosal lesions has been associated with less favorable longer-term clinical outcomes in Crohn’s disease,^19^^,^^20^ the prognostic utility of MRI-based scores has been less well established. METRIC-EF included 194 patients newly diagnosed with Crohn’s disease, where MRI was performed soon after diagnosis and patients prospectively followed up over 5 years to assess disease progression. Addition of MRI activity scores, including sMaRIA, to a multivariable clinical model did not aid prediction of future “disabling” Crohn’s disease.^21^ Data from PROFILE, however, rather contradict this: all nine patients who required abdominal surgery during the 48 weeks of the PROFILE trial had abnormal MRIs at baseline, with sMaRIA scores that were significantly higher than patients who did not go on to require surgery (Figure 3). Importantly, no such correlation was seen between ileo-colonoscopy findings/severity of SES-CD scores and need for abdominal surgery. Our findings are consistent with results from a previous observational study of 112 patients, where MRI but not endoscopic abnormalities were associated with later need for surgery.^22^

The frequency with which the Montreal classification of disease behavior based on clinical findings typically available at diagnosis (history, examination, endoscopy, and blood tests) had to be revised with the addition of MRI results is instructive: 6% of cases of “clinical” B1 were reclassified as radiological B2 or B3. The consequences of misclassification were particularly evident for patients managed with the step-up treatment strategy: 4/12 such patients, with B2 or B3 disease evident on their baseline MRI, required surgery over the next 48 weeks. Conversely top-down therapy effectively “rescued” patients who had B2 or B3 disease evident at index MRI: no patients in this group came to surgery.

Despite growing interest in non-invasive imaging as a potential endpoint for Crohn’s disease clinical trials, sMaRIA demonstrated limited utility as a tool for assessing treatment response in the PROFILE trial dataset. Indeed, had sMaRIA scores been used in PROFILE, the significant difference in outcomes between “step-up” and “top-down” treatment arms seen on ileo-colonoscopy would have been missed^13^ (Figure 4;  Figure S2). As indicated above, this may in part be explained by the fact that many patients had only mild or inactive disease at the end of the PROFILE trial. Nonetheless, in clinical trials the ability to distinguish active versus inactive mucosal disease and treatment response versus non-response with high fidelity is paramount. Previous studies on the utility of MRI and sMaRIA in assessing therapy outcomes compared findings on scans performed before and after treatment. Two such studies showed that the sMaRIA score correlated with ileo-colonoscopy findings and could detect changes after treatment—but in aggregate they included just 87 patients.^11^^,^^23^ Our study was much larger and had a different design, using only end-of-trial investigations to compare between treatment arms. This, plus the fact that the PROFILE end-of-trial cohort had a much higher proportion of patients in remission, may help explain differences in conclusions.

Our study has several key strengths. It included the largest cohort of patients with Crohn’s disease undergoing paired ileo-colonoscopies and MRI scans reported to date. As most had a B1 phenotype, this enabled robust comparison of the ability of each investigation to detect active mucosal inflammatory disease. The fact that our study was rooted in the PROFILE randomized controlled trial also allowed comparison of the utility of the different imaging modalities in assessing response to treatment in Crohn’s disease. We focused on the end-of-trial investigations, when treatments were stable and the interval between studies was short, and excluded those done at baseline where there was a relatively long time gap between ileo-colonoscopy and MRI during which treatment changed significantly. The process for the selection and training of central readers for both ileo-colonoscopy and radiology was rigorous.^14^^,^^15^

Our study also had limitations. Since a high proportion of patients at week 48 in the PROFILE trial were in remission, the study population might be atypical for the population of patients in whom MRIs have historically been undertaken. In real-world practice such patients are usually symptomatic, and clinicians are keen to exclude strictures or fistulae. In our cohort, only a small proportion of patients had such complications, perhaps giving a falsely poor impression of the overall performance of MRI. Part of our study focused on terminal ileal sub-scores to enable direct comparison between sMaRIA and SES-CD in an area of the bowel that both typically visualize well. It must be acknowledged that such segment-specific sub-scores have not been prospectively validated. While MRIs deemed technically inadequate were excluded, the 1% deemed “sub-optimal,” for example for reasons of inadequate small bowel distension, were included as this was deemed by our radiologists to better reflect real-world practice. Finally, as a potential challenge to regulators who place significant emphasis on endoscopic end-points, it could be argued that the prognostic significance of endoscopically active inflammation/ulceration in the absence of radiologically significant disease is unknown.

Our study raises important questions for future research. Although we focused on evaluation of MRI and comparisons to ileo-colonoscopy, IUS is increasingly being adopted around the world and has shown high reliability and low inter-observer variability in the context of Crohn’s disease.^24^ Indeed, IUS has potential advantages over MRI in being potentially more available, repeatable, and possible to perform as a point-of-care test with high levels of patient acceptability. With the growing evidence supporting use of IUS in Crohn’s disease, it will be important for future research studies to similarly evaluate how its scores correlate with SES-CD in detecting and quantifying inflammatory disease, and whether IUS scores could be used as an endpoint in clinical trials.

Our findings support the notion that MRI and ileo-colonoscopy are complementary imaging modalities and that one cannot displace the other in their established roles in clinical practice or clinical trials. MRI has clear advantages in assessing transmural disease and complications (strictures and fistulae)—including in the proximal small bowel, which is not readily accessible endoscopically. Ileo-colonoscopy provides a more accurate assessment of mucosal inflammation, which may itself guide decisions around treatment escalation/de-escalation.

## 5. Conclusions

There is continuing interest in the extent to which ileo-colonoscopy can be replaced by non-invasive imaging for objective inflammatory assessment and evaluation of treatment response in Crohn’s disease. Here we have shown that the sMaRIA score from MRI had only moderate ability to detect inflammatory disease activity or treatment response evident on ileo-colonoscopy SES-CD scoring. These findings have potential implications for both clinical practice and clinical trials. Our conclusion is that ileo-colonoscopy and MRI should remain complementary studies, reflecting their different respective strengths and weaknesses.

## Supplementary Material

jjag056_Supplementary_Data

## Data Availability

Access to the study data and other materials is managed by the Cambridge University Hospitals R&D Study Review Committee. Please contact the corresponding author.
