# Supplementary material for: Magnetic resonance imaging-based simplified MaRIA scores are associated with future surgery in Crohn’s disease, but modest correlation with ileo-colonoscopic inflammation limits their utility in clinical trials: results from the PROFILE trial
Source: J Crohns Colitis. 2026 May 19;20(5):jjag056. doi: 10.1093/ecco-jcc/jjag056 (PMC13195631; doi:10.1093/ecco-jcc/jjag056)

**Supplementary Appendix**

A total of 18 scans were evaluated in two reading rounds by five consultant gastrointestinal radiologists. The readers were blinded to patient symptoms and endoscopy findings, with a consensus meeting held between the two rounds. Inter-reader reliability was assessed using the intraclass correlation coefficient (ICC). To further validate this in a clinical trial setting, 71 scans from the PROFILE trial were independently double-read and ICC calculated. After two rounds of training, the central reader group demonstrated good inter-reader reliability, with ICC values above 0.70 and the lower bound of the 95% confidence interval exceeding 0.5.[^15^](https://www.zotero.org/google-docs/?ZdyfW7)

**Supplementary Table 1. Correlation of sMaRIA and SES-CD by Binary Classification of Inactive vs Active at week 48**

| **sMaRIA**  **SES-CD** | **Inactive** | **Active** | **Total** |
| --- | --- | --- | --- |
| **Inactive** | 88 | 25 | 113 |
| **Active** | 41 | 66 | 107 |
| **Total** | 129 | 91 | 220 |

**Supplementary Table 2. SES-CD and sMaRIA severity category thresholds.**

| **SES-CD score** | **Category** | **sMaRIA score** |
| --- | --- | --- |
| SES-CD ≤ 2 | Inactive | sMaRIA = 0 |
| 3<SES-CD<6 | Mild | sMaRIA = 1 |
| 7<SES-CD<15 | Moderate | sMaRIA = 2 |
| SES-CD ≥ 16 | Severe | sMaRIA ≥ 3 |

**Supplementary Table 3. Correlation of sMaRIA and SES-CD by Disease Location at start and end of trial.**

| Disease Location | N | Spearman’s Correlation coefficient  (Start of Trial) |
| --- | --- | --- |
| Ileal | 83 | 0.235245 |
| Ileocolonic | 145 | -0.100872 |
| Colonic | 70 | 0.216175 |

| Disease Location | N | Spearman’s Correlation coefficient  (End of Trial) |
| --- | --- | --- |
| Ileal | 83 | 0.574616 |
| Ileocolonic | 145 | 0.407400 |
| Colonic | 70 | 0.451432 |

**Supplementary Table 4. Correlation Between sMaRIA Parameters and SES-CD Scores by Intestinal Segment**

| Segment | sMaRIA Parameter | N | Pearson r | P-value | Strength |
| --- | --- | --- | --- | --- | --- |
| Terminal Ileum | Mural oedema | 220 | 0.470 *** | <0.001 | Moderate |
| Terminal Ileum | Wall thickening | 220 | 0.471 *** | <0.001 | Moderate |
| Terminal Ileum | Fat stranding | 220 | 0.399 *** | <0.001 | Moderate |
| Terminal Ileum | Ulcer | 220 | 0.300 *** | <0.001 | Moderate |
| Right Colon | Mural oedema | 220 | 0.255 *** | <0.001 | Weak |
| Right Colon | Wall thickening | 220 | 0.283 *** | <0.001 | Weak |
| Right Colon | Fat stranding | 220 | 0.119 | 0.0592 | Weak |
| Right Colon | Ulcer | 220 | 0.088 | 0.1607 | Negligible |
| Transverse Colon | Mural oedema | 220 | 0.394 *** | <0.001 | Moderate |
| Transverse Colon | Wall thickening | 220 | 0.360 *** | <0.001 | Moderate |
| Transverse Colon | Fat stranding | 220 | 0.129 * | 0.0393 | Weak |
| Transverse Colon | Ulcer | 220 | 0.129 * | 0.0393 | Weak |
| Left Colon | Mural oedema | 220 | 0.103 | 0.1006 | Weak |
| Left Colon | Wall thickening | 220 | 0.074 | 0.2387 | Negligible |
| Left Colon | Fat stranding | 220 | 0.073 | 0.2492 | Negligible |
| Left Colon | Ulcer | 220 | N/A | N/A | N/A |
| Rectum | Mural oedema | 220 | 0.281 *** | <0.001 | Weak |
| Rectum | Wall thickening | 220 | 0.281 *** | <0.001 | Weak |
| Rectum | Fat stranding | 220 | 0.328 *** | <0.001 | Moderate |
| Rectum | Ulcer | 220 | N/A | N/A | N/A |

*N = number of paired observations. Significance levels: *** p<0.001, ** p<0.01, * p<0.05, ns = not significant. Correlation strength: |r| ≥0.50 strong, 0.30-0.49 moderate, 0.10-0.29 weak, <0.10 negligible. N/A indicates no ulcers detected in this segment (Left Colon and Rectum).*

**Supplementary Table 5. Disease classification pre- and post-MRI and baseline sMaRIA and SES-CD scores of patients requiring surgery.**


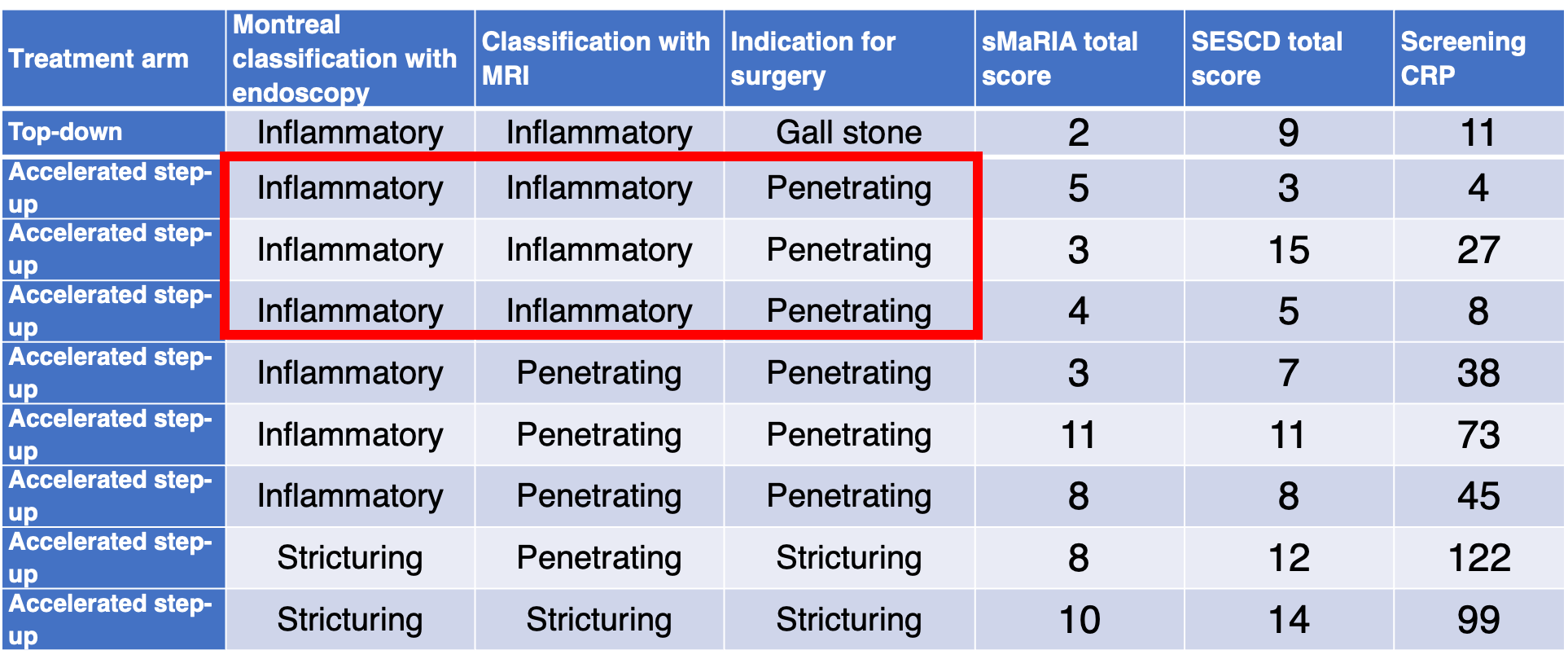


**Supplementary Table 6. Reclassification of disease phenotype by pre and post MRI and surgical outcomes**

This table shows how the initial Montreal classification pre-MRI (based on history, examination, blood tests and ileo-colonoscopy) changed once additional information from MRI was available (‘post-MRI’) for patients in the PROFILE ‘step-up’ and ‘top-down’ treatment arms.


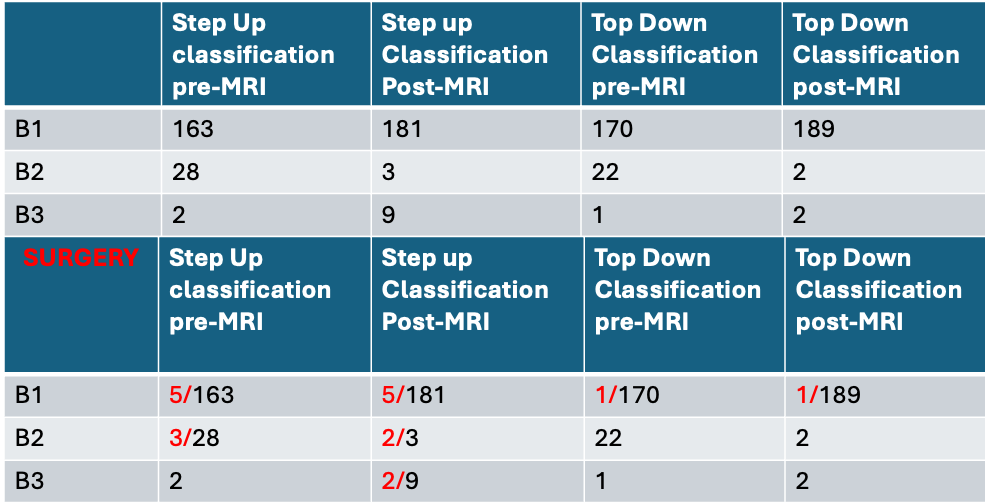


**Supplementary Figure 1.**

**A) Endoscopic remission defined by absence of moderate and large ulcers (i.e. SES-CD maximum ulcer subscore score ≤1) and B) Endoscopic remission defined SES-CD ulcer subscore score of 0 and C) MRI-defined remission defined by a sMaRIA ulcer subscore of 0.**


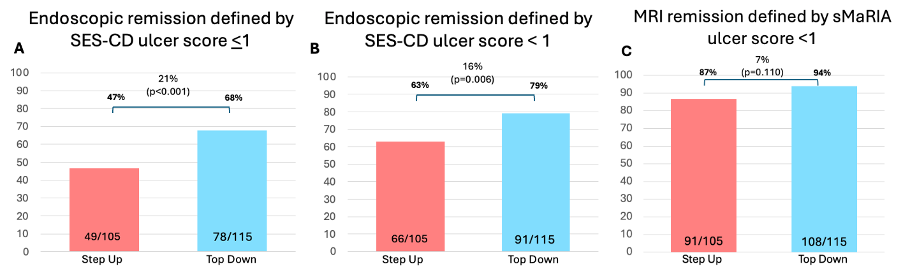

Supplement: jjag056_Supplementary_Data [file jjag056_supplementary_data.zip › Supplementary Appendix 1.docx]
