# Supplementary figures and images for: Magnetic resonance imaging-based simplified MaRIA scores are associated with future surgery in Crohn’s disease, but modest correlation with ileo-colonoscopic inflammation limits their utility in clinical trials: results from the PROFILE trial
Source: J Crohns Colitis. 2026 May 19;20(5):jjag056. doi: 10.1093/ecco-jcc/jjag056 (PMC13195631; doi:10.1093/ecco-jcc/jjag056)

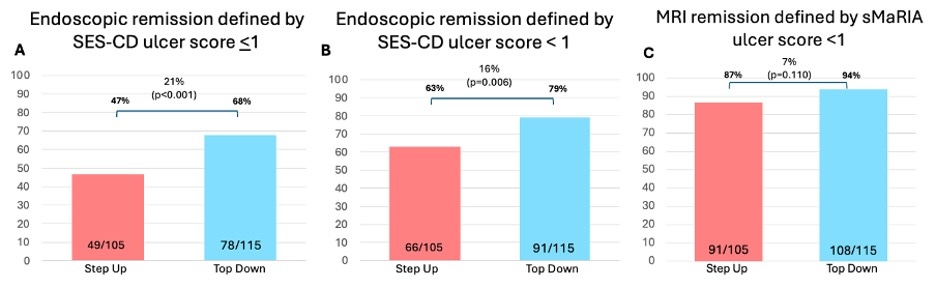

Supplement: jjag056_Supplementary_Data [file jjag056_supplementary_data.zip › Supplementary Fig 1.jpg]

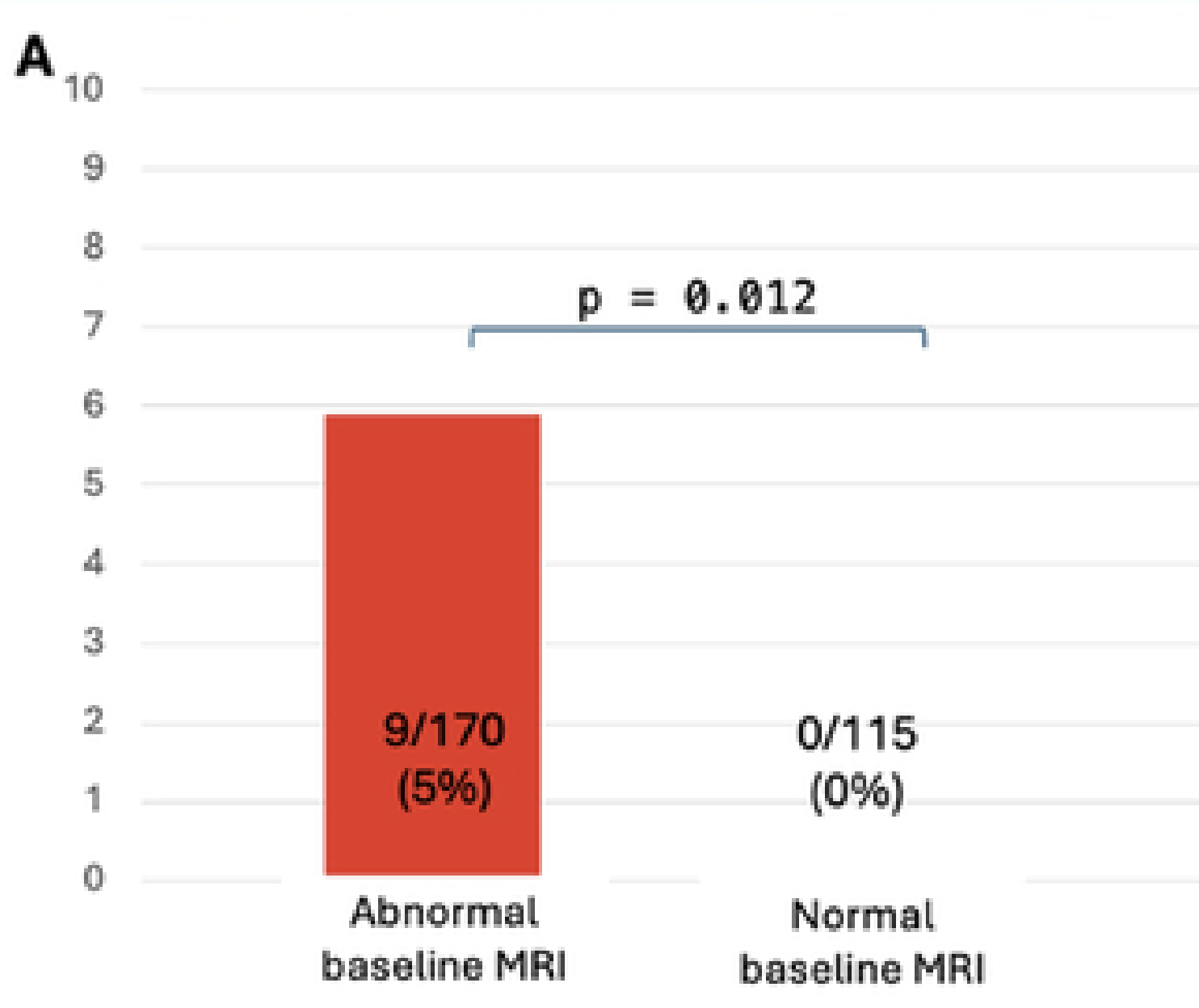

Supplement: jjag056_Supplementary_Data [file jjag056_supplementary_data.zip › Fig 3A.png]
